# Authors’ Reply to: Toward a Better Understanding of Quality Social Connections. Comment on “Quality Social Connection as an Active Ingredient in Digital Interventions for Young People With Depression and Anxiety: Systematic Scoping Review and Meta-analysis”

**DOI:** 10.2196/37440

**Published:** 2022-03-11

**Authors:** Lindsay H Dewa, Lily Roberts, Emma Lawrance, Hutan Ashrafian

**Affiliations:** 1 Institute of Global Health Innovation Imperial College London London United Kingdom; 2 School of Public Health Imperial College London London United Kingdom; 3 Mental Health Innovations London United Kingdom

**Keywords:** mental health, digital interventions, young people, quality social connection, depression, anxiety, systematic review, meta-analysis, patient and public involvement, mobile phone

We welcome feedback from the authors Deng and Qin through their comment [1] on our paper “Quality Social Connection as an Active Ingredient in Digital Interventions for Young People With Depression and Anxiety: Systematic Scoping Review and Meta-analysis” [2]. They suggest that two included studies [3,4] should not be included in our review as individual studies as data extraction and quality assessment of both studies is the same, and that this impacts the quality of the meta-analysis. We have the following response. First, our review is scoping in nature, therefore we included and extracted any study that matched our inclusion criteria and appropriately extracted each study separately. Second, both studies have different designs and methodologies, and obtain different outcomes. For example, one study is a co-designed study, and the other is a qualitative study; therefore, neither study should be included in the meta-analysis itself. Third, these studies are included and discussed in the review in relation to indicators of quality social connection within digital interventions and each study finding is different. In this case, different data are presented and referenced in relation to distinct digital intervention mechanisms that facilitate quality social connection and they are appropriately presented separately. For example, the importance of anonymity is mentioned in one but not the other. Finally, we have already discussed the statistical and methodological variation within our scoping review as a potential limitation.

Thank you for considering our response to the comment.
